# Tetrahedral DNA Nanostructures: A Structural-Family-Aware Review from Computational Design and Biological Stability to Translational Nanomedicine

**DOI:** 10.3390/nano16191227

**Published:** 2026-09-29

**Authors:** Paulina G. Rosales-Pérez, J. Alejandro Morales, Luis J. Figueroa-Yáñez

**Affiliations:** 1 University Center for Biological and Agricultural Sciences (CUCBA), Universidad de Guadalajara , Guadalajara 45200, Mexico; 2 Industrial Biotechnology Unit, Center for Research and Assistance in Technology and Design of the State of Jalisco, A.C. (CIATEJ), Zapopan 44270, Mexico; 3 Biodigital Innovation Lab, Translational Bioengineering Department, Centro Universitario de Ciencias Exactas e Ingenierías (CUCEI), Universidad de Guadalajara, Guadalajara 44430, Mexico

**Keywords:** tetrahedral DNA nanostructures, tetrahedral framework nucleic acids, DNA origami, computational design, biological stability, aptamer targeting, drug delivery, translational nanomedicine

## Abstract

DNA has evolved from a carrier of genetic information into a programmable material for constructing nanoscale architectures with precise structural and functional control. Among these systems, tetrahedral DNA nanostructures (TDNs) comprise at least two experimentally distinct structural families: small scaffold-free tetrahedra assembled from a few synthetic oligonucleotides, often termed tetrahedral framework nucleic acids (tFNAs), and larger scaffolded or wireframe tetrahedral DNA-origami objects. This review integrates assembly principles, computational and sequence-level design, physicochemical stability, fabrication, structural validation, biological performance, and translational evidence while keeping these two families analytically separate. For few-strand TDNs, particular attention is given to ionic conditions, pH, temperature, serum and nuclease exposure, cellular uptake, targeting aptamers, and delivery of drugs and regulatory nucleic acids; scaffolded tetrahedral origami is discussed where its routing, mechanics, manufacturing, or larger spatial capacity are directly relevant. Recent in vivo studies provide evidence for tissue distribution, transdermal delivery, and short-term biocompatibility of selected few-strand TDN formulations, but the translational evidence remains predominantly preclinical. Rather than positioning TDNs as universally superior nanocarriers, this review emphasizes architecture-specific evidence, quantitative experimental conditions, and the limits of transferring conclusions between structural families. Progress toward translational nanomedicine will require validated design rules, matched-condition benchmarking, pharmacokinetic and biodistribution studies, scalable manufacturing, standardized quality attributes, and rigorous safety assessment.

## 1. Introduction

DNA origami was formally introduced in 2006 by Paul Rothemund of the California Institute of Technology (Caltech) in a landmark article published in Nature. The method transformed DNA nanotechnology by enabling a long single-stranded viral DNA molecule, typically the M13mp18 genome, to be folded with hundreds of short synthetic oligonucleotides known as staples into arbitrary two-dimensional shapes, including stars and smiley faces, with nanometer-scale precision [1].

In 2009, scaffolded DNA origami expanded into three dimensions through multilayer and honeycomb-lattice designs [2,3]. Douglas and colleagues subsequently introduced caDNAno, a computer-aided design platform for routing scaffold and staple strands in lattice-based architectures, substantially simplifying the design of complex three-dimensional DNA-origami objects [4].

Over the following decade, DNA origami evolved from geometric demonstrations toward increasingly functional architectures. Liu et al. reported crystalline two-dimensional DNA-origami arrays [5], and Zhao et al. described hierarchical assembly of preformed origami tiles into larger super-origami structures [6]. Broader applications now include biosensing and delivery-oriented DNA nanomaterials [7], while RNA-origami approaches enable cotranscriptional folding and extend programmable nucleic acid nanotechnology into synthetic biology [8].

DNA tetrahedra constitute a distinct early three-dimensional motif within structural DNA nanotechnology. Goodman et al. showed that few-strand tetrahedra can self-assemble rapidly with near-quantitative yield into rigid, chiral structures [9]. Subsequent studies established that tetrahedral DNA cages can enter mammalian cells without conventional transfection reagents [10,11], and geometry-dependent comparisons later showed a strong effect of corner angle on cellular entry [12]. Their compact, addressable architecture has consequently been developed for biosensing and drug-delivery applications [13,14,15]. Nuclease resistance, however, is design- and condition-dependent rather than an intrinsic consequence of the tetrahedral shape [16,17].

Given the potential of these nanostructures, this review analyzes recent advances in programmable design, structural stability under challenging biological conditions, and the diverse biotechnological applications of DNA tetrahedra, with particular attention to their current role as precision tools in nanomedicine.

DNA nanotechnology is one of the most dynamic areas of contemporary nanomedicine, with direct implications for drug delivery systems, molecular diagnostics, and gene therapy. Within this field, the DNA tetrahedron (TDN) has gained substantial translational relevance, not because it is the most complex architecture, but because it combines several otherwise difficult-to-reconcile attributes: straightforward synthesis, triangulation-derived mechanical rigidity, stability in biologically challenging environments, and precise functionalization at each vertex [13,15,18,19].

Despite the rapidly expanding literature, evidence remains fragmented. Structural stability, computational design, and biomedical applications are often discussed separately rather than integrated within a unified critical framework. Moreover, systematic comparisons between tetrahedra and other DNA nanostructures under equivalent physiological conditions remain limited, complicating evidence-based experimental design [16,19,20,21,22,23].

This review is therefore motivated by the need to consolidate current knowledge without treating all tetrahedral DNA architectures as interchangeable. Throughout this article, “few-strand TDN/tFNA” refers to small scaffold-free tetrahedra assembled from a few synthetic oligonucleotides, whereas “scaffolded/wireframe tetrahedral origami” refers to larger objects that require a long scaffold, many staple strands, or wireframe routing. Unless direct comparative evidence is available, conclusions about design rules, ionic stability, manufacturing, cellular uptake, biodistribution, or therapeutic performance are not transferred from one family to the other. The review integrates sequence-level design, architecture-appropriate computational tools, physicochemical stability, fabrication, validation, biological performance, and translational evidence within a family-aware design-to-translation framework. 

Recent reviews already cover important parts of the tFNA field, but with different organizing questions. Gu et al. focus explicitly on current understanding and translational prospects of four-strand tFNAs; Fan et al. emphasize engineering strategies and biomedical innovation; He et al. synthesize disease-oriented and precision-biomedicine applications; and Su et al. center on rational structural design and structure–property relationships [24,25,26,27]. The present review differs in four specific ways: it separates few-strand TDNs from scaffolded/wireframe tetrahedral origami throughout the analysis; maps computational tools to the structural family for which they are actually appropriate; tabulates primary experimental conditions rather than comparing outcomes detached from buffer, serum, size, and readout; and links those design choices to a tiered validation and translational-evidence framework. Table 1 summarizes these differences in scope without implying that the approaches are mutually exclusive.

As a narrative Review, the literature considered herein was selected from peer-reviewed primary studies and recent reviews addressing TDN design, stability, fabrication, computational analysis, biological performance, and biomedical applications. PubMed and publisher/DOI-level searches were used to identify relevant literature, with emphasis on studies providing direct experimental or mechanistic evidence. Studies of other DNA architectures were included when they provided relevant comparative or conceptual context.

## 2. What Exactly Is DNA Origami?

Although the term “DNA origami” was introduced in 2006, the conceptual foundations of structural DNA nanotechnology date to Seeman’s 1982 proposal to use branched DNA junctions as programmable components for three-dimensional networks [28]. Naturally occurring junctions can be conformationally labile when sequence symmetry permits branch migration; Kallenbach, Ma, and Seeman subsequently demonstrated sequence-designed immobile junctions [29].

To suppress branch migration, Seeman and colleagues used asymmetric sequence design to create immobile DNA junctions [29]. These concepts enabled progressively more complex DNA assemblies, while double-crossover motifs later provided robust structural units for periodic DNA architectures [30,31]. Historical reviews summarize the subsequent progression from branched junctions to polyhedral and crossover-based DNA assemblies [7,30].

Rothemund defined scaffolded DNA origami as folding a long single-stranded scaffold into a predetermined two-dimensional shape with many short staple strands; the original implementation used the M13mp18 scaffold [1]. Douglas et al. extended this strategy to compact three-dimensional DNA-origami objects [3]. Functional DNA nanodevices were subsequently engineered to sense molecular inputs and expose or release payloads, including the logic-gated nanorobot demonstrated by Douglas, Bachelet, and Church [32]. These developments established a route from static scaffolded architectures to responsive DNA nanodevices [32,33].

Douglas also developed caDNAno, a visual honeycomb-lattice interface that allows users to route scaffold and staple strands across a geometric grid. Subsequent efforts sought to create more precise structures without being constrained by naturally occurring scaffolds. The Bathe group introduced algorithms that derive scaffold routing and staple sequences from a target 3D geometry, leading to DAEDALUS and related engineering platforms for automated wireframe design. In parallel, Hendrik Dietz and colleagues established biotechnological methods for producing DNA scaffolds with customized lengths and sequences using modified phage systems, thereby overcoming limitations imposed by the native M13 genome [4,34,35,36,37].

## 3. Thermodynamics of Self-Assembly: From Stochastic Interactions to Programmable Folding

DNA nanostructure assembly is programmable because sequence complementarity and scaffold/staple routing constrain the set of accessible base-pairing interactions. Three-dimensional origami studies demonstrated reproducible folding of highly connected architectures [3], while top-down wireframe design showed that target geometry can be converted algorithmically into scaffold routing and staple sequences [35]. Assembly fidelity nevertheless remains sensitive to architecture, sequence design, annealing conditions, and the analytical method used to detect misfolding [3,35].

Self-assembly also requires controlled thermal conditions. In many DNA-origami protocols, the mixture is heated to approximately 65 °C to disrupt pre-existing structures and then cooled gradually so that each staple can locate its intended binding site. The optimal annealing program is design-dependent, however, and small scaffold-free tetrahedra may use substantially shorter or different protocols. Inadequate annealing can produce misfolded intermediates and aggregates because strands may hybridize with unintended partners or become kinetically trapped [3,15].

## 4. Why the Tetrahedron Among So Many Geometries?

The DNA tetrahedron occupies a central position in biomedical nanotechnology not because it is the most complex architecture or has the greatest cargo capacity but because few-strand designs combine rapid synthesis, triangulation-derived rigidity, surface addressability, and efficient cellular uptake [9,12,13,15]. Direct comparisons across all geometries remain limited; consequently, these advantages should be interpreted as design-dependent trends rather than universal geometric rules.

One of the best-documented geometric effects is cell entry. Peng et al. showed that uptake differed among tetrahedra, triangular prisms, and cubes and linked these differences to corner angle and scavenger–receptor interactions [12]. Separately, tetrahedral DNA cages have been shown to enter mammalian cells without conventional transfection reagents [10,11]. Together, these observations support a role for architecture in cellular internalization, while not implying that tetrahedral geometry will dominate across every cell type or formulation [12,13].

After membrane contact, TDN internalization has been reported to involve caveolin-associated, clathrin-independent uptake in the live-cell system examined by Liang et al., followed by intracellular trafficking toward lysosomal compartments [11]. The relative contribution of endocytic routes can vary with cell type, TDN size, sequence, surface functionalization, and culture conditions [18,22]. Lysosomal accumulation alone does not demonstrate efficient endosomal escape or nuclear delivery; those endpoints should therefore be validated independently when nuclear-targeting or other trafficking modules are incorporated [22].

## 5. Stability of DNA Tetrahedra

### 5.1. Ionic Stability

Low divalent-cation concentrations in biological media can challenge DNA nanostructure integrity because electrostatic repulsion between negatively charged phosphate backbones is commonly screened with millimolar Mg^2+^ during folding and storage [20,21]. The estimated reference range for circulating ionized Mg^2+^ in healthy adults is approximately 0.40–0.68 mmol/L [38], substantially below the Mg^2+^ concentrations used in many DNA-origami protocols. Structures that depend strongly on high divalent-cation concentrations may therefore destabilize when transferred to physiological media [20,21].

Selected DNA nanostructures, including tetrahedral architectures, can be assembled or maintained under alternative ionic conditions, but the result depends strongly on architecture, ion identity, and concentration [20,39,40]. Rodriguez et al. directly demonstrated cation-dependent assembly across several DNA motifs and showed that monovalent and divalent ions are not interchangeable for tetrahedron formation under matched conditions [40]. Lower local helix density and reduced electrostatic crowding remain plausible contributors to ionic tolerance in some open architectures; reduced electrostatic repulsion has been demonstrated for selected triangulated wireframe systems [41], but this mechanism has not been established as a general causal explanation for canonical few-strand TDNs.

### 5.2. Stability Across pH Conditions

When evaluating pH stability, the relevant question is not simply whether a structure disassembles, but how its geometry changes across the sequence of environments encountered during delivery. Extracellular physiological fluids are near neutral, whereas the extracellular tumor microenvironment is commonly reported as moderately acidic, approximately pH 6.0–6.9 [42]. Endosomal–lysosomal compartments become more acidic, with lysosomes typically near pH 4.5–5.5 [43]. These environments should therefore not be represented by a single acidic interval. For TDNs specifically, Wang et al. demonstrated pH-dependent shrinkage and stretching of a tetrahedral construct [44]. The extent and reversibility of such structural responses should be interpreted as sequence-, junction-, exposure-time-, and ionic-condition-dependent rather than as a universal property of all TDNs.

Near neutral pH, DNA nanostructures are generally compatible with extracellular and cytoplasmic conditions, although persistence remains sensitive to ionic composition and nuclease activity [20,21]. Under increasingly alkaline conditions, disruption of base pairing can promote denaturation, but no universal alkaline threshold can be assigned to TDNs because architecture, buffer composition, sequence, and analytical readout differ across studies [20,21]. Results from other DNA architectures should therefore not be converted into a direct ranking of tetrahedral alkaline stability. Heavy-metal-mediated stabilization has been explored as one strategy for extending DNA-origami stability under demanding conditions [45].

### 5.3. Thermal Stability

The thermal stability of DNA tetrahedra depends on sequence composition, junction topology, base stacking, ligand binding, ionic conditions, and the specific architecture tested. Carter et al. found that, despite sequence differences among individual edges, the investigated tetrahedral complex displayed cooperative thermal denaturation, with the monitored edges melting at comparable temperatures rather than behaving as six independently melting arms. Binding of the minor-groove ligand Hoechst 33258 increased the thermal stability of the tetrahedral complex. More recently, Punnoose et al. demonstrated that terminal base-stacking interactions can be deliberately tuned to modulate tetrahedron stability; the magnitude of the melting-temperature shift and the ranking of specific stacking pairs are properties of the tested designs and should not be generalized to all TDNs [46,47].

Direct quantitative ranking of the thermal stability of tetrahedra, planar origami, cubes, prisms, and other DNA nanostructures remains difficult because available studies differ substantially in sequence composition, buffer conditions, Mg^2+^ concentration, strand concentration, topology, surface interactions, and analytical methodology. Melting temperatures reported for different structures under non-equivalent conditions should therefore not be interpreted as head-to-head evidence of geometric superiority. Thermal stability is better treated as a design- and condition-dependent property, and future comparative work should benchmark alternative architectures under matched experimental conditions [21,46,47].

A related translational consideration is nuclease resistance. DNA nanostructure topology and compaction can alter enzyme accessibility relative to free linear DNA [17], but protection is not absolute. For tetrahedra, degradation is sensitive to size and nuclease identity [16], while serum stability also depends on the complete architecture and formulation [17,23]. Cross-architecture rankings should therefore be made only under matched conditions.

**Figure 1:**
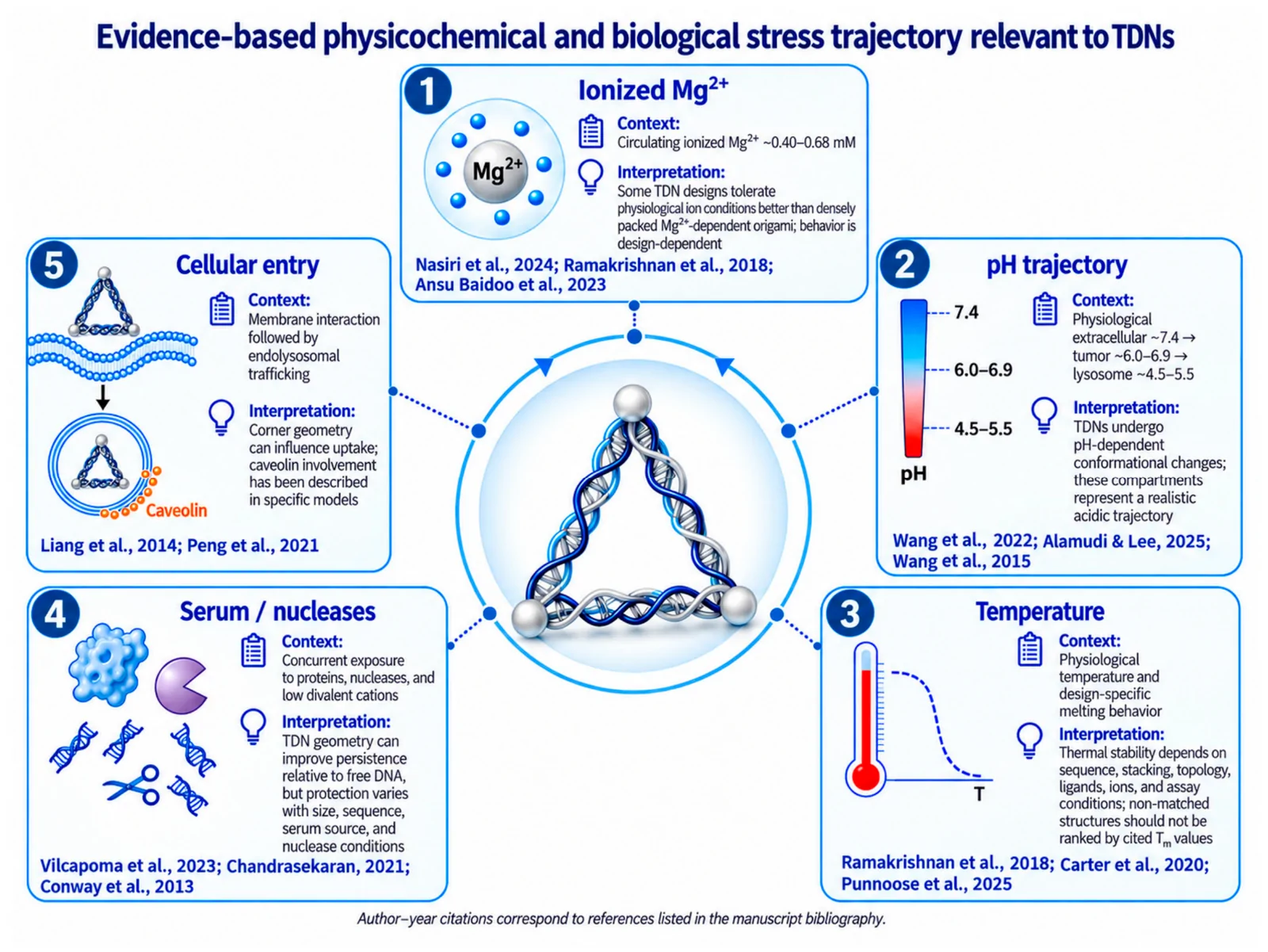
Evidence-based physicochemical and biological stress trajectory relevant primarily to few-strand tetrahedral DNA nanostructures (TDNs/tFNAs). The schematic summarizes key conditions encountered during biological transit, including ionized magnesium, pH transitions, physiological temperature, serum and nuclease exposure, and cellular entry. Numerical values are shown only when supported by defined physiological or experimentally reported ranges, whereas structural and biological outcomes remain dependent on sequence, size, formulation, ionic environment, and experimental conditions. The scheme is not intended to imply direct head-to-head superiority over other DNA nanostructures or to extrapolate these behaviors to scaffolded/wireframe tetrahedral origami without direct evidence. Solid arrows indicate the sequential biological trajectory, dotted connectors link each stress condition to the central TDN, and the color-coded panels and schematic shapes distinguish the principal physicochemical and biological environments encountered during transit. The ionized-magnesium context is supported by Refs. [20,21,38]; pH conditions and pH-dependent TDN responses by Refs. [42,43,44]; thermal behavior by Refs. [21,46,47]; serum and nuclease exposure by Refs. [16,17,23]; and cellular entry by Refs. [11,12]. TDN, tetrahedral DNA nanostructure; tFNA, tetrahedral framework nucleic acid; Mg^2+^, magnesium ion. Original graphical representation created by the authors with graphical assistance from ChatGPT (GPT-5.6 Sol, OpenAI, 2026), with OpenAI image-generation tools.

In biological environments, ionic strength, temperature, protein adsorption, nuclease activity, and pH act in sequence and combination rather than independently. During biological transit, TDNs encounter low divalent-cation concentrations, serum proteins, and active nucleases, followed by compartment-specific pH changes after cellular uptake. Their ability to retain integrity across this succession of stresses underpins their value as targeted-delivery platforms and provides the basis for the biomedical applications discussed below (Figure 1) [17,18,21,23].

## 6. In Silico Design of TDNs: A Structural-Family-Specific Workflow

Computational workflows for tetrahedral DNA nanostructures (TDNs) should be selected according to structural family rather than applied as a universal six-step pipeline. Two distinct design problems are considered here: (i) small scaffold-free tetrahedra assembled from four to a few synthetic oligonucleotides, which dominate the biomedical literature discussed in Section 7, Section 8 and Section 9, and (ii) larger scaffolded or wireframe tetrahedral origami, which require explicit routing of a long scaffold and many staple strands. For the first family, manual domain design and sequence-level thermodynamic screening are usually the most directly relevant computational steps, whereas lattice routing and automated wireframe CAD are generally unnecessary. For the second family, CAD, routing, mechanical simulation, and structural refinement become central [4,48,49,50,51,52,53,54,55,56,57,58,59,60].

Lattice-based origami CAD, including caDNAno and scadnano, is therefore mainly applicable to scaffolded tetrahedral origami. These tools route scaffold and staple strands across honeycomb or square lattices and are valuable when the intended tetrahedral object is constructed from multihelix bundles or face-forming origami panels. They are not normally required for canonical four-strand TDNs, in which the six edges are specified directly by complementary sequence domains [4,51].

Lattice-free or semi-manual CAD, including Tiamat and vHelix, provides greater geometric freedom for large three-dimensional objects. Tiamat supports interactive three-dimensional editing, whereas vHelix converts polygonal or polyhedral meshes into DNA routing. Their principal use is in scaffolded, wireframe, or otherwise geometrically elaborate tetrahedral objects rather than in few-strand biomedical TDNs [52,53].

Automated wireframe design should likewise be interpreted as a scaffolded-origami branch. DAEDALUS provides top-down sequence design for three-dimensional polyhedral wireframe DNA origami; METIS addresses two-dimensional wireframe/honeycomb-edge designs; and ATHENA supports rapid prototyping of arbitrary two- and three-dimensional wireframe objects through a graphical interface and simulation-compatible output [35,54,55].

Integrative three-dimensional modeling tools such as Adenita and ENSnano support interactive construction, visualization, and manipulation of modular DNA nanostructures [56,57]. For small scaffold-free TDNs, these platforms are optional rather than prerequisite; for larger hybrid or scaffolded tetrahedra, they can assist spatial assembly planning and handoff to downstream simulation.

Mechanical and dynamic simulation spans several resolution levels. CanDo estimates equilibrium shape and flexibility for DNA-origami-like structures, while oxDNA/oxView and mrDNA provide coarse-grained or multiresolution access to conformational dynamics, fraying, interduplex angles, junction flexibility, and trajectories [49,58,59,60]. At finer resolution, all-atom molecular dynamics can explicitly represent DNA, solvent, counterions, base stacking, and junction microenvironments. Atomistic simulations have demonstrated that DNA-origami structures can deviate locally from idealized CAD geometries because of steric, electrostatic, and solvent-mediated forces, and they are particularly appropriate when the scientific question concerns ion-specific, sequence-specific, or junction-level behavior rather than only global shape [61]. Because of their computational cost, all-atom simulations are best used selectively and, when possible, after lower-resolution screening. Recent work on nanopore simulation further illustrates how all-atom trajectories can be coupled to experimentally measurable ionic-current observables [62].

Sequence thermodynamics is the computational layer most directly applicable to few-strand TDNs. NUPACK can evaluate intended hybridization, competing complexes, strand competition, and unintended secondary structures, whereas ViennaRNA is useful when RNA-containing modules, hairpins, or responsive RNA cargoes are incorporated. These tools do not replace three-dimensional simulation, but they provide a practical pre-synthesis filter for both scaffold-free and scaffolded tetrahedral systems [48,50].

**Figure 2:**
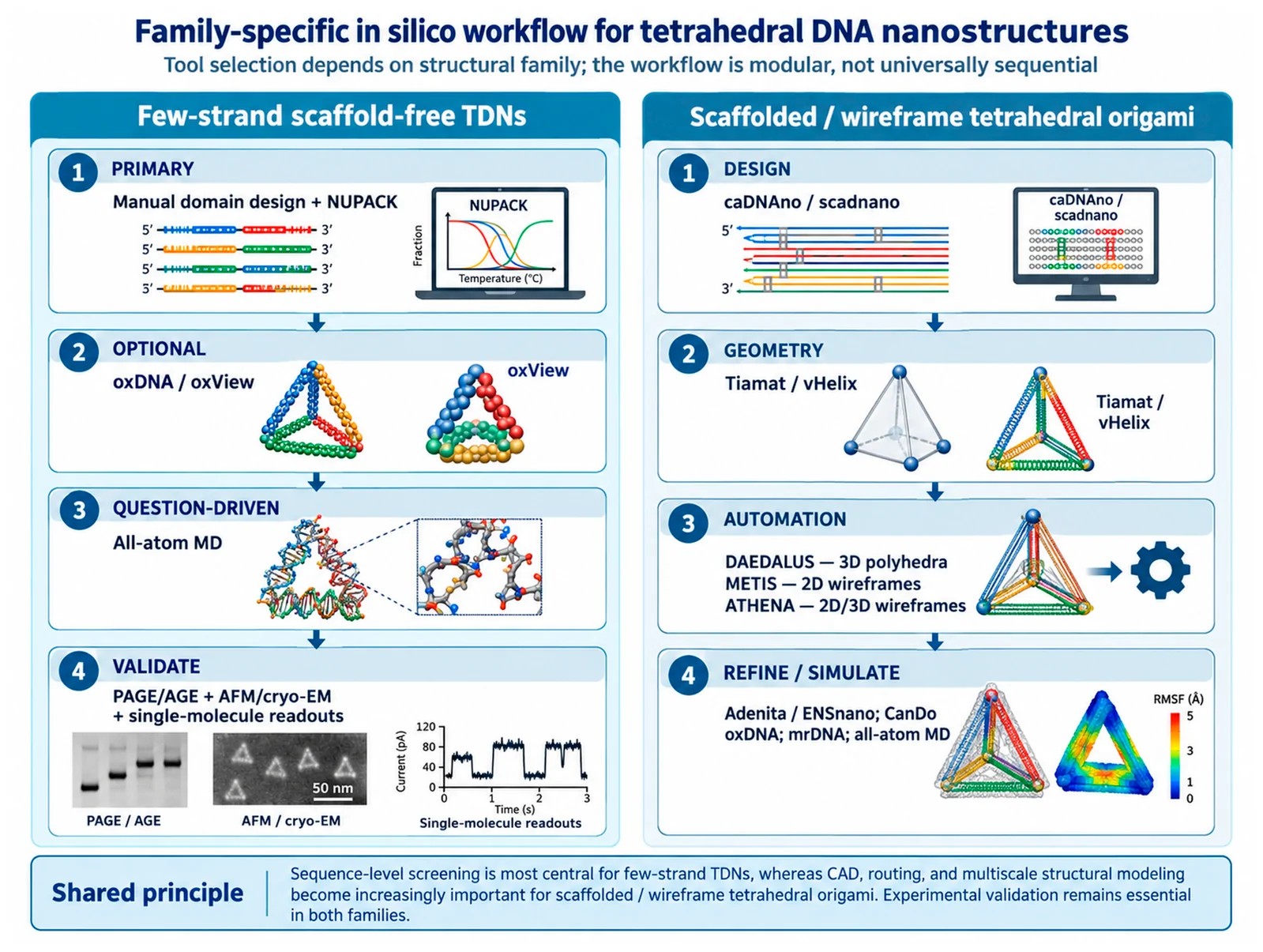
Structural-family-specific in silico workflow for tetrahedral DNA nanostructures. Few-strand scaffold-free TDNs are primarily designed at the sequence/domain level, whereas scaffolded or wireframe tetrahedral origami generally require CAD, routing, and multiscale structural modeling. The computational workflow is modular and question-driven rather than universally sequential, and experimental validation remains essential in both structural families. Arrows and connecting lines indicate progression and relationships among workflow stages; boxed modules denote individual computational or experimental steps, and the color-coded branches distinguish few-strand scaffold-free TDNs from scaffolded/wireframe tetrahedral origami. Original graphical representation created by the authors with graphical assistance from ChatGPT (GPT-5.6 Sol, OpenAI, 2026), with OpenAI image-generation tools.

Worked example—few-strand TDN: A canonical four-oligonucleotide tetrahedron such as the Goodman et al. design [9] illustrates the appropriate branch of the workflow. The experimentally demonstrated tetrahedra used four strands and regular 20 bp edges (with related variants containing 20 and 30 bp edges), assembled in 10 mM Tris/5 mM MgCl2 by heating to 95 °C and cooling rapidly to 4 °C, with reported near-quantitative assembly. Retrospectively, a modern computational workflow for this structural family would begin with manual domain assignment, use NUPACK to screen unintended strand interactions, optionally use oxDNA when global flexibility or junction behavior is a design variable, and reserve all-atom MD for questions that specifically require ion-, base-stacking-, or junction-level resolution. The computational predictions would then be tested experimentally by native electrophoresis and direct structural imaging. This example is intended as a mapping of an experimentally validated TDN onto the relevant computational layers, not as a claim that all of these software tools were used in the original 2005 study. The structural-family-specific workflow is summarized in Figure 2, and the applicability, inputs, outputs, and experimental validation routes of the principal computational tools are compared in Table 2.

## 7. From Digital Design to Experimental Fabrication

Two broad structural families should be distinguished. Small scaffold-free tetrahedra are assembled from a few synthetic strands and are widely used when rapid synthesis and biomedical functionalization are priorities [9,15,63]. Larger tetrahedral origami objects use a long scaffold and many staples and can provide larger cavities or more extensive spatial engineering capacity [64]. More elaborate scaffolded or wireframe systems bring additional routing and manufacturing requirements [34,35,36,37,41].

Small tetrahedra are typically designed from oligonucleotides containing complementary domains that form the six edges and are assembled by one-pot annealing. This route remains widely used because it is rapid and sufficiently robust for delivery, targeting, and regenerative studies. Functional extensions, pH-sensitive modules, aptamers, intercalating drugs, and bioswitchable components can be added without altering the core assembly logic. Single-strand assembly routes have also been reported, expanding the possibilities for replicable design [9,15,63].

Larger tetrahedra and molecular containers generally use scaffolded-origami strategies. A long single-stranded scaffold, often derived from M13mp18, is folded by dozens or hundreds of shorter staples. This approach supports larger objects and more precisely defined cavities but entails greater design, purification, and manufacturing complexity [36,37,64].

Short scaffold-free strands and staple oligonucleotides are generally produced commercially by solid-phase phosphoramidite synthesis. Long scaffolds exceeding several kilobases are rarely synthesized chemically in their entirety; instead, they are typically generated biologically using phage or bacterial/phagemid systems and subsequently purified. Scalable biotechnological production of DNA origami and custom-length scaffolds has already been demonstrated [36,37].

When natural scaffolds are avoided and all strands are synthetic, two major strategies are available. The first is the simple few-strand tetrahedron, which is particularly relevant to biomedical applications. The second uses single-stranded tiles or fully addressable scaffold-free architectures to build larger wireframes from repeated synthetic modules. Fully synthetic systems eliminate dependence on a biological scaffold and permit tighter sequence control, but cost and purification burden increase with strand number [41,63].

## 8. Biomedical Applications and Stimulus-Responsive Release Systems

Biomedical applications of DNA tetrahedra are most clearly organized according to cargo type and activation mechanism. TDNs can transport small molecules, oligonucleotides, miRNA, siRNA, and diagnostic probes, while aptamers can be displayed at defined vertices or edges as molecular-recognition elements. Relative to free nucleic acid strands, they provide spatial organization, multivalency, cellular uptake, and a programmable surface on which each vertex can serve as a defined functional address. These features underpin applications in active targeting, RNA delivery, aptamer display, diagnostics, and cancer therapy [14,15,19,65,66,67,68].

In chemotherapy, representative cargoes include doxorubicin and other intercalating molecules. TDNs can combine drug loading, molecular recognition, and controlled release within a single architecture. AS1411-functionalized tetrahedra, for example, can recognize nucleolin-expressing tumor cells and promote selective doxorubicin delivery with pH-responsive release [67]. Broader cancer-delivery literature supports related aptamer-functionalized TDN systems [68].

Recent work in RNA delivery and gene regulation has focused on dynamic and bioswitchable systems. Such designs can transport miRNA or siRNA and release the cargo in response to defined stimuli, with potential applications in wound healing, tissue regeneration, and gene-expression control. This direction is particularly important because it connects digital structural design with measurable functional outcomes in cells and tissues [65,69].

In immunotherapy, DNA tetrahedra can serve as multivalent platforms for organizing immunostimulatory or antigenic components, an approach reviewed across DNA-nanostructure immunotherapy systems [70]. TDNs have also been directly investigated for induction of immune tolerance; in non-obese diabetic mouse models, Gao et al. reported tFNA-mediated immunomodulation and prevention of type 1 diabetes onset [71].

**Figure 3:**
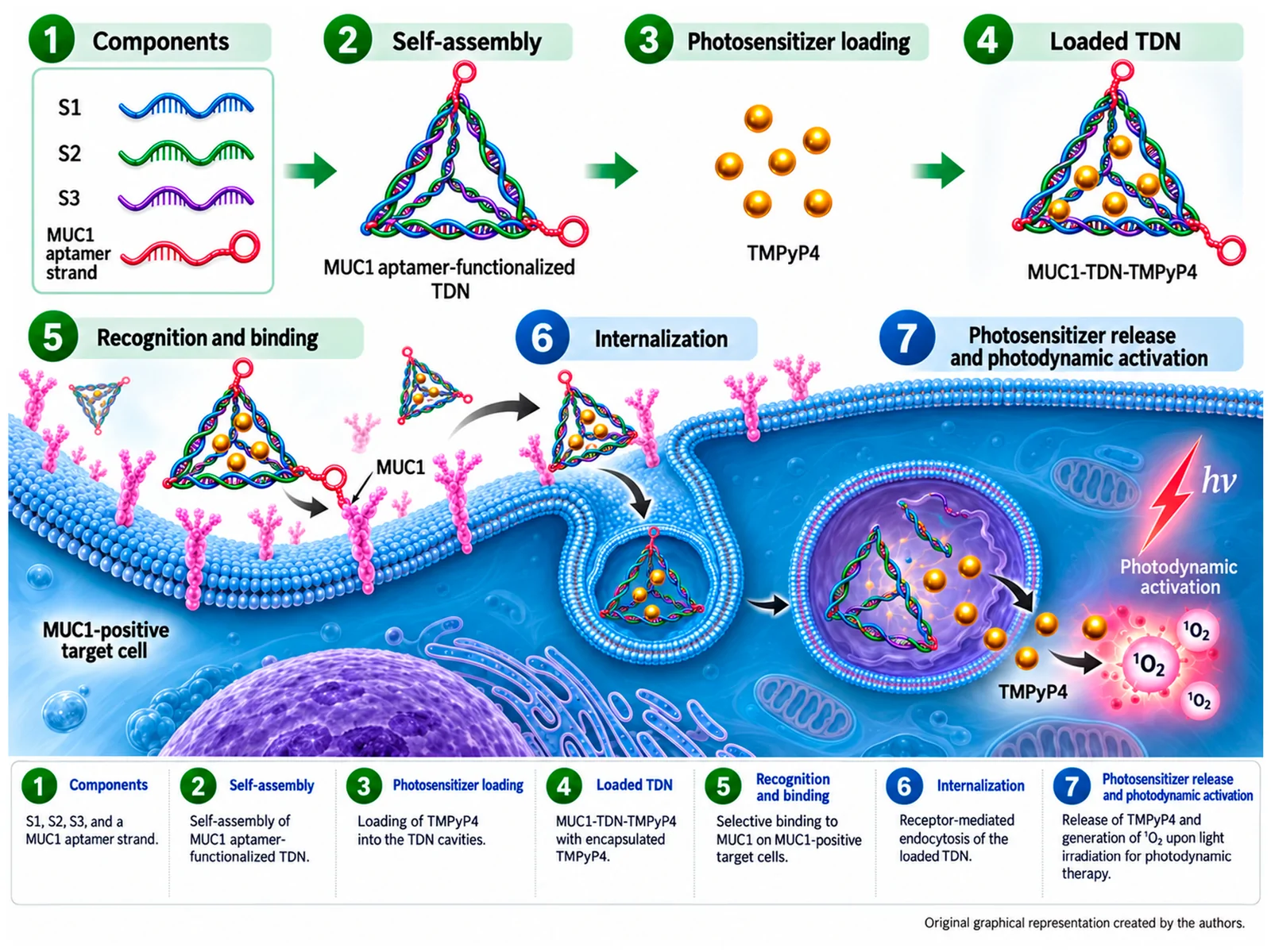
Conceptual workflow of a MUC1-targeted tetrahedral DNA nanostructure (TDN) for photodynamic therapy. A MUC1 aptamer is displayed on the TDN as the molecular-recognition element, while TMPyP4 is incorporated as the photosensitizer cargo. Following selective binding to MUC1-positive target cells, the loaded TDN is internalized by endocytosis, enabling intracellular TMPyP4 release and subsequent light-triggered photodynamic activation through singlet-oxygen generation. The schematic summarizes the targeting, internalization, cargo-release, and photodynamic-activation strategy reported for MUC1 aptamer-functionalized TDNs and is not a reproduction or graphical adaptation of published artwork [72]. Arrows indicate the sequential progression from TDN assembly and cargo loading to molecular recognition, cellular internalization, cargo release, and photodynamic activation; numbered and color-coded schematic modules distinguish the principal stages of the process. Original graphical representation created by the authors with graphical assistance from ChatGPT (GPT-5.6 Sol, OpenAI, 2026), with OpenAI image-generation tools. The authors reviewed and edited the final figure to ensure scientific accuracy.

Recognition aptamers are among the most important strategies for achieving selective delivery. They can be positioned at TDN vertices or edges to recognize specific cell-surface receptors and enhance uptake by target cells. In cancer applications, this approach can direct nanostructures toward cells that overexpress selected markers while potentially reducing accumulation in nontarget tissues [67,68]. A representative photodynamic-therapy implementation uses a MUC1 aptamer displayed on TDNs to target MUC1-positive cells, while TMPyP4 serves as the photosensitizing cargo [72] (Figure 3).

Beyond acting as passive carriers, tetrahedra can be engineered as activatable systems using recognition aptamers, blocking or logic strands, pH-sensitive structural elements, or strand-displacement/bioswitch modules [32,44,65,66,67]. In these systems, the trigger and release mechanisms are design-specific; therefore, activation, intracellular trafficking, and functional cargo release should be validated in the relevant biological model [22,65,66,67].

In regenerative medicine, tetrahedral framework nucleic acids have been investigated as functional biomaterials that modulate proliferation, migration, differentiation, and tissue repair. These properties have motivated their evaluation in wound healing, bone regeneration, oral tissues, and other regenerative contexts [73].

## 9. Experimental Validation of DNA Tetrahedra

Confirmation of correct TDN assembly requires a tiered analytical strategy rather than a single technique, because each method addresses a different structural or functional attribute [15,20].

Native polyacrylamide gel electrophoresis (PAGE) or agarose electrophoresis provides the first level of validation. Successful assembly is typically indicated by a discrete band with mobility distinct from that of free strands or incomplete intermediates. This rapid and accessible approach provides an initial assessment of assembly yield, gross purity, and completeness of assembly and remains a standard first-line method in TDN studies [9,15,64].

Direct imaging provides morphology information, but the structural claim supported by each method should be stated carefully. Atomic force microscopy (AFM) can show surface-adsorbed particles with dimensions and projected shapes consistent with tetrahedral assembly, but mica adsorption can flatten or distort three-dimensional objects; accordingly, AFM alone does not establish solution-state three-dimensional geometry [9,15,64]. For scaffolded wireframe tetrahedra, single-particle cryogenic electron microscopy (cryo-EM) with three-dimensional reconstruction can more directly characterize solution-like architecture and quantify deviations from the design model [35].

Dynamic light scattering (DLS) provides complementary information on hydrodynamic size and aggregation and has been used in tetrahedral DNA characterization workflows [15,64]. Reported TDN dimensions span from the sub-10-nm scale of early few-strand tetrahedra to much larger scaffolded objects and should therefore be compared with the dimensions predicted for the specific construct rather than treated as a universal size criterion [9,15,16,64]. Circular dichroism (CD) spectroscopy provides complementary information on DNA helical conformation and folding state [15,20].

Structural characterization must be complemented by testing under biologically relevant conditions. TDNs should be incubated in serum, at physiological pH, and in the presence of nucleases, with integrity monitored over time relative to unexposed controls. Such assays are essential for anticipating in vivo behavior and should precede advanced cellular or cargo-delivery studies [17,21,23].

When a tetrahedron carries a functional payload, such as doxorubicin, miRNA, or siRNA, or is equipped with a targeting aptamer, validation must establish structural integrity, correct cargo incorporation, retention of recognition activity, and stimulus-responsive release where applicable [15,67,68]. Fluorescence-based assays can quantify loading and monitor release kinetics, while binding assays can assess ligand or aptamer functionality. Confocal microscopy and flow cytometry can then evaluate cellular uptake and help distinguish nonspecific association from receptor-mediated recognition [22,67,68].

Single-molecule structural readouts provide an additional validation level between ensemble characterization and high-resolution imaging. Nanopore-based interrogation is particularly attractive because individual DNA nanostructures can be detected in solution through transient ionic-current blockades without fluorescent labels. For TDNs, target-induced deformation has been read out with a glass nanopore as a change in current-pulse amplitude, demonstrating that conformational transitions of a tetrahedral construct can generate single-molecule electrical signatures [74]. Nanopore observables are sensitive to molecular volume, topology, flexibility, and translocation dynamics, and their interpretation can be strengthened by molecular simulation; all-atom approaches are especially useful when relating local molecular structure and ion distributions to measured current signals [62,74]. Single-molecule methods should therefore be considered complementary to PAGE, AFM, cryo-EM, DLS, CD, and ensemble fluorescence rather than replacements for them.

## 10. Current Translational Evidence, Limitations, and Open Questions

For few-strand TDN/tFNA systems, the translational evidence is currently preclinical rather than clinical. In vivo work has nevertheless moved beyond cell-culture proof of concept. Differently sized tFNAs have been compared for endocytosis and whole-animal biodistribution, with size-dependent behavior and detectable access to multiple organs, including the brain, reported in mice [75]. Framework nucleic acids have also been used as programmable transdermal carriers in animal models, demonstrating tissue delivery rather than only in vitro uptake [76]. These studies establish biologically relevant distribution and delivery, but they do not yet define human pharmacokinetics, repeated-dose safety, dose–exposure relationships, or clinical efficacy.

Safety evidence is encouraging but incomplete. A systematic mammalian-cell study reported no major adverse cellular response under the tested conditions [77], and multiple disease models support therapeutic activity of functionalized tFNA constructs. However, these data remain formulation-, dose-, route-, and model-specific. Major unresolved biological issues include nuclease degradation, variable serum stability, immunological effects, off-target accumulation, renal clearance, and long-term toxicity [17,20,22,24]. Manufacturing constraints are architecture-specific: few-strand TDNs are affected chiefly by oligonucleotide synthesis, modification, purification, and batch control [15,24], whereas scaffolded origami additionally requires reproducible scaffold/staple production and batch-level structural quality control [36,37].

The evidence base also differs sharply by structural family. Most biodistribution, cellular-uptake, regenerative, and therapeutic studies concern small scaffold-free TDNs/tFNAs, whereas manufacturing and CAD literature often concerns larger scaffolded or wireframe DNA origami. These bodies of evidence should not be merged without direct experimental justification. The TDN literature rarely provides matched head-to-head comparisons across architectures, doses, routes, buffers, serum conditions, and analytical endpoints. Table 3 makes this heterogeneity explicit by presenting experimental conditions alongside the reported outcomes. Accordingly, translational claims in this review are restricted to the structural family and experimental context actually tested.

To facilitate cross-study comparison under explicitly defined experimental conditions, Table 3 compiles representative structural dimensions, assembly and ionic conditions, stability assays, analytical readouts, and cellular or functional outcomes from primary and protocol-level TDN studies discussed throughout this review.

## 11. Discussion

The central message emerging from the current literature is not that all tetrahedral DNA architectures form a single interchangeable platform. For small scaffold-free TDNs/tFNAs, the most developed biomedical evidence concerns rapid assembly, cellular uptake, functionalization, cargo delivery, and regenerative or therapeutic effects. For scaffolded/wireframe tetrahedral origami, the strongest evidence concerns geometric programmability, routing, structural mechanics, and larger spatial design capacity. The two families share nucleic acid programmability but differ in strand architecture, manufacturing burden, relevant computational tools, and the maturity of biological evidence; conclusions should therefore remain family-specific unless direct comparisons are available.

More importantly, the accumulated evidence indicates that geometry alone is insufficient to predict performance. Ionic composition, pH, nuclease exposure, serum interactions, strand sequence, size, and surface functionalization can substantially alter both structural persistence and cellular behavior [16,17,20,21,22,23,44,47]. Stability should therefore be interpreted not as an intrinsic attribute of the tetrahedral shape but as an emergent property of the complete nanoformulation as it moves through a defined biological trajectory. This distinction is critical because the stresses encountered from administration to intracellular trafficking are sequential, heterogeneous, and mechanistically coupled rather than independent experimental variables.

The rapid expansion of computational design tools further changes the conceptual status of TDN development. CAD platforms, sequence-thermodynamic analysis, coarse-grained simulation, and, for selected mechanistic questions, all-atom molecular dynamics increasingly enable structural liabilities to be identified before synthesis and reduce empirical iteration [4,48,49,50,51,52,53,54,55,56,57,58,59,60,61,62]. Their greatest value, however, lies not in replacing experimentation but in establishing an iterative design–build–test–learn framework in which simulation, physicochemical characterization, single-molecule readouts, and biological performance continuously inform one another. In this sense, TDNs provide a tractable model system for converting DNA nanotechnology from a structure-building discipline into a progressively more predictive engineering field.

The principal translational challenge is consequently no longer whether TDNs can be assembled or functionalized, but whether their performance can be reproduced across laboratories, batches, biological models, and manufacturing scales. For few-strand systems, priority areas include defined critical quality attributes, matched-condition benchmarking, biologically realistic stability testing, biodistribution, and intracellular trafficking [15,19,20,22]. For scaffolded or wireframe systems, scalable scaffold/staple production and batch-specific structural quality control are additional requirements [36,37]. The decisive threshold for the field will be reached when structural programmability is accompanied by reproducible biological behavior.

## 12. Future Directions

Future work should prioritize the conversion of empirical observations into transferable design rules. This will require prospective studies in which sequence composition, tetrahedral size, vertex geometry, ligand density, cargo loading, and physicochemical conditions are varied systematically and evaluated using matched analytical criteria. Linking these datasets to coarse-grained simulation and sequence-level thermodynamic modeling could enable computational predictions to be calibrated against measurable structural and biological outcomes rather than used only as pre-synthesis visualization tools [19,20,47,49,50].

A second priority is biological standardization. TDN stability and function should be evaluated under increasingly realistic conditions that reproduce serum exposure, nuclease activity, physiologically relevant ionic environments, endosomal acidification, and cell-type-specific trafficking. Parallel measurements of uptake, endosomal escape, intracellular persistence, biodistribution, pharmacokinetics, immunological effects, and off-target accumulation will be essential for distinguishing efficient cellular entry from therapeutically meaningful delivery [11,16,17,21,22,23,65,66].

Finally, translational development will depend on manufacturing strategies that connect nanoscale design with reproducible product quality. For few-strand TDNs, development should address oligonucleotide quality, purification, batch-to-batch structural verification, and predefined critical quality attributes [15,19]. Scaffolded origami additionally requires scalable scaffold production and staple/routing quality control [36,37]. Comparative benchmarking against established nanocarriers should be integrated early rather than added after proof of concept. Advances in active targeting and stimulus-responsive systems further suggest that future TDN platforms will be most competitive when structural programmability is used to solve a clearly defined biological problem rather than to maximize architectural complexity [65,66,67,72].

## 13. Conclusions

Tetrahedral DNA nanostructures encompass structurally distinct families that should not be collapsed into a single evidentiary category. Small scaffold-free TDNs/tFNAs are experimentally accessible and have accumulated substantial biomedical evidence, whereas scaffolded/wireframe tetrahedral origami offers broader geometric engineering capacity but follows different design and manufacturing logic. Their shared importance lies in programmable nucleic acid architecture, but the strength and type of evidence supporting stability, biological performance, and translation are family-specific.

The evidence reviewed here also makes clear that successful translation will not be determined by architecture alone. Biological performance emerges from the interaction between sequence, geometry, formulation, environmental stability, intracellular trafficking, and manufacturing quality. Accordingly, future progress should prioritize reproducible design rules, standardized characterization under biologically relevant conditions, quantitative comparison with established delivery systems, and scalable production compatible with clinical-grade requirements.

TDNs therefore represent more than a specific nanocarrier geometry: they provide a useful testbed for understanding how digital nucleic acid design can be converted into reliable function in complex biological environments. Their long-term translational value will depend on whether this programmability can be transformed into predictable, reproducible, and application-specific performance.

## Figures and Tables

**Table 1:** Scope comparison between the present review and recent reviews of tetrahedral DNA nanostructures. The comparison highlights differences in structural scope, organizing emphasis, treatment of computational design, quantitative synthesis of experimental conditions, and translational focus. The table summarizes coverage and perspective and is not intended to rank review quality. The arrow (→) denotes conceptual progression between successive stages of the design-to-translation framework.

Review	Primary Structural Scope	Main Organizing Emphasis	Computational Design Treated Explicitly	Quantitative Experimental-Condition Synthesis	Translation Emphasis
Gu et al., 2025 [24]	Primarily few-strand tFNA	Biological properties, applications, clinical-translation prospects	Limited	No matched-condition primary-study table	Strong
Fan et al., 2025 [25]	Primarily few-strand tFNA	Engineering, delivery, intrinsic therapeutic activity	Limited	No matched-condition primary-study table	Challenges/future perspectives
He et al., 2026 [26]	Primarily few-strand tFNA	Disease mechanisms and precision biomedicine	Limited	No matched-condition primary-study table	Clinical potential and barriers
Su et al., 2026 [27]	Primarily few-strand tFNA	Rational design and structure–property relationships	Yes, conceptually	Not organized as matched-condition benchmarking	Translation hurdles
Present Review	Few-strand TDN/tFNA plus explicitly separated scaffolded/wireframe tetrahedral origami	Family-aware design → stability → validation → preclinical translation	Yes; tool applicability mapped by family	Yes; primary conditions and readouts tabulated	Existing preclinical evidence separated from future requirements

**Table 2:** Applicability of computational design, modeling, and simulation tools across few-strand and scaffolded/wireframe tetrahedral DNA nanostructures. The table summarizes the typical input, primary output, most relevant structural family, and representative downstream experimental validation or linkage for each computational tool or modeling layer.

Tool/Layer	Typical Input	Primary Output	Most Relevant Structural Family	Representative Downstream Experimental Validation or Linkage
caDNAno/scadnano [4,51]	Long scaffold; staples; lattice geometry	Scaffold/staple routing	Scaffolded tetrahedral origami	AGE/PAGE; AFM/TEM/cryo-EM
Tiamat/vHelix [52,53]	3D geometry or polyhedral mesh	Editable 3D DNA model/routed architecture	Scaffolded or wireframe tetrahedra	AFM/TEM/cryo-EM
DAEDALUS [35]	Target 3D polyhedral mesh	Scaffold routing + staple sequences	3D scaffolded polyhedra	AGE; TEM/cryo-EM
METIS [54]	Target 2D wireframe/honeycomb-edge design	Automated sequence/routing design	2D wireframes; not canonical few-strand TDNs	AGE; AFM/TEM
ATHENA [55]	2D or 3D wireframe geometry	GUI-based sequence/routing output	2D/3D wireframe origami	AGE; AFM/TEM/cryo-EM
Adenita/ENSnano [56,57]	DNA modules; optional protein/aptamer components	Integrated 3D model	Hybrid/scaffolded; optional for small TDNs	Imaging; binding/functional assays
CanDo [58]	Origami routing/topology	Equilibrium shape and flexibility	Mostly scaffolded origami	AFM/TEM/cryo-EM dimensions
oxDNA/oxView [49]	Sequence/topology; initial 3D coordinates	Coarse-grained trajectories and flexibility	Both families; optional for small TDNs	FRET, AFM, cryo-EM, thermal/structural assays
mrDNA [59]	Large DNA structure	Multiresolution structural dynamics	Large/scaffolded systems	Imaging and mechanical observables
All-atom MD [61,62]	Atomic DNA model, solvent, ions	Atomic trajectories; ion/base/junction behavior	Both families when local resolution is required	Structural comparison to experimental geometry; nanopore ionic-current observables
NUPACK [50]	Oligonucleotide sequences and concentrations	Hybridization equilibria; off-target complexes	Especially few-strand TDNs; also modules in origami	Native-gel, melting, or hybridization assays in downstream validation
ViennaRNA [48]	RNA sequences/modules	RNA secondary structure and thermodynamics	RNA-containing TDN modules	Native-gel and structure/function assays for RNA-containing modules

**Table 3:** Representative quantitative and experimental parameters reported in primary and protocol-level studies of tetrahedral DNA nanostructures. The table summarizes TDN architecture and size, assembly or ionic conditions, serum and stability assays, structural readouts, and cellular or functional outcomes. Numerical values are included only when directly reported in the cited source; otherwise, the experimental focus or absence of a standardized quantitative value is indicated. Because experimental conditions differ substantially among studies, the table is intended to support contextual comparison rather than direct head-to-head ranking. In the assembly/annealing conditions, the arrow (→) indicates a temperature transition from the initial temperature to the final temperature.

Primary Study	TDN Structure/Size	Assembly or Ionic Conditions	Serum/Stability Condition and Outcome	Structural Readout	Cell Model/Uptake or Functional Metric
Goodman et al., 2005 [9]	4 oligonucleotides; regular 20 bp edges; variants with 20/30 bp edges	10 mM Tris, 5 mM MgCl_2_; 95 → 4 °C in 30 s	Not a serum study; assembly yield reported > 95% for standard preparation	Native PAGE; AFM; ligation/restriction mapping	No cell model; structural benchmark
Walsh et al., 2011 [10]	Small four-strand tetrahedral cage; ~7 nm class	Synthetic oligonucleotide self-assembly	Not a dedicated serum-half-life study; intracellular structural integrity was observed for at least 48 h after transfection	Electrophoresis; confocal microscopy; flow cytometry; FRET	Mammalian cells; uptake without transfection reagent; intracellular localization assessed
Vilcapoma et al., 2023 [16]	Three tetrahedra; 13–20 bp edge lengths	Matched sequences differing in tetrahedron size	FBS biostability; 20 bp tetrahedron degraded ~4× faster than 13 bp tetrahedron	Gel-based degradation assays; DNase I and T5 exonuclease challenges	No uptake study
Peng et al., 2021 [12]	Tetrahedron compared with triangular prism and cube; geometry/corner-angle series	Matched nanostructure comparison	Not primarily a serum-stability study	Structural characterization plus cellular uptake assays	Tetrahedron showed highest uptake among the compared geometries; corner angle correlated with entry
Liang et al., 2014 [11]	Small TDN suitable for live-particle tracking	Fluorescently labeled TDN	Intracellular integrity/trafficking evaluated rather than serum half-life	Single-particle tracking; live-cell fluorescence	Mammalian cells; caveolin-associated, clathrin-independent entry reported in the tested system
Rodriguez et al., 2023 [40]	Self-assembled DNA nanostructures including tetrahedral architectures	Multiple mono- and divalent cation conditions	Not a serum study; assembly yield and nuclease resistance were strongly cation-dependent	Gel and structural characterization	No cell model; ion dependence quantified experimentally
Wang et al., 2015 [44]	TDN and i-motif-connected TDN	pH 8.5 versus pH 4.5	Not a serum study; reversible/condition-dependent shape response	Structural dimension/conformation measurements	No cell model; pH-dependent shrink/stretch
Carter et al., 2020 [46]	4-oligonucleotide TDN with site-specific fluorescent probes	Thermal melting with/without Hoechst 33258	Not a serum study; ligand increased thermal stability	Fluorescence melting analysis	No cell model; cooperative edge melting
Punnoose et al., 2025 [47]	Tetrahedra assembled through sticky-end/base-stacking interfaces	Terminal base-stacking variants; thermal series	Not a serum study; single stacking change shifted Tm by up to ~10 °C	Thermal stability assays; all-atom MD for selected stacking interfaces	No cell model
Ke et al., 2009 [64]	Scaffolded DNA-origami tetrahedral container; ~54 nm edge	Long scaffold + multiple staples	Not a serum study	Gel electrophoresis; AFM; TEM; DLS	No cell model; large scaffolded benchmark
Li et al., 2009 [63]	Single-strand replicable tetrahedral nanostructure	Single-strand folding architecture	Not a serum study	Electrophoresis and structural characterization	No cell model
Li et al., 2025 [65]	Few-strand TDN carrying bioswitchable miRNA modules	Sequence-programmed cargo/loading architecture	Protocol-level study; stability and stimulus-dependent functional release are validated within the defined workflow	Gel/fluorescence/functional validation	Cell/tissue models; miRNA release and biological response
Tian et al., 2023 [66]	Dynamic TDN delivery framework with active targeting modules	Stimulus-responsive TDN assembly	Protocol-level study; cargo retention/release is condition- and construct-dependent rather than a universal half-life	Electrophoresis, imaging, loading/release assays	Target-cell uptake and on-site cargo release
Lyu et al., 2024 [69]	tFNA-based BiRDS carrying three miRNAs	Nucleic acid core preserving tetrahedral framework	High stability reported; storage/biological stability evaluated in the study	Gel and fluorescence-based characterization	Cell and tissue permeability; wound-healing functional outcomes
Gao et al., 2021 [71]	Tetrahedral framework nucleic acid immunomodulatory platform	Few-strand tFNA formulation	Not a serum-stability study; in vivo immunomodulatory efficacy was evaluated in the disease model	Structural and immunological assays	Autoimmune diabetes model; immune-tolerance endpoints
Meng et al., 2021 [72]	MUC1-aptamer-functionalized TDN loaded with TMPyP4	Targeting aptamer + photosensitizer cargo	Stimulus/photodynamic functionality rather than serum benchmark	Gel/fluorescence/cellular imaging	MUC1-positive breast cancer cells; targeted uptake and photodynamic response
Wang et al., 2019 [74]	Hairpin-containing TDN used as nanopore signal amplifier	Target binding deforms/stretches one TDN edge	No serum study	~20-nm glass nanopore; single-molecule ionic-current pulses	No cell model; target-induced current-pulse amplitude change

## Data Availability

No new data were created or analyzed in this study. Data sharing is not applicable to this article.

## Abbreviations

The following abbreviations are used in this manuscript:

2D	Two-dimensional
3D	Three-dimensional
AFM	Atomic force microscopy
AGE	Agarose gel electrophoresis
CAD	Computer-aided design
CD	Circular dichroism
cryo-EM	Cryogenic electron microscopy
DLS	Dynamic light scattering
DNA	Deoxyribonucleic acid
FRET	Förster resonance energy transfer
GUI	Graphical user interface
MD	Molecular dynamics
miRNA	MicroRNA
MUC1	Mucin 1
PAGE	Polyacrylamide gel electrophoresis
RNA	Ribonucleic acid
siRNA	Small interfering RNA
TDN	Tetrahedral DNA nanostructure
TEM	Transmission electron microscopy
tFNA	Tetrahedral framework nucleic acid
TMPyP4	5,10,15,20-Tetrakis(1-methyl-4-pyridinium) porphyrin
